# MiR-33a-5p in stored red blood cells regulates genes of innate immune response and promotes inflammation

**DOI:** 10.18632/aging.205925

**Published:** 2024-06-25

**Authors:** Jingrui Zhang, Dan Zhang, Jing Zhao, Wei Zheng

**Affiliations:** 1 Department of Transfusion Medicine, General Hospital of Northern Theater Command, Shenyang 110000, China

**Keywords:** blood stored, red blood cell, miRNA, macrophage, inflammatory

## Abstract

Background and Objectives: Blood transfusion is a common therapeutic procedure in hospitalized patients. Red blood cell (RBC) units undergo various biochemical and morphological changes during storage (storage lesion). miRNAs have been studied intensively regarding cellular metabolic processes, but the effect of miRNAs on blood storage is not well defined.

Materials and Methods: We performed bioinformatics analysis on the public data set of miRNA expression of RBC based on R language, and performed the Kyoto encyclopedia of genes and genomes (KEGG) enrichment analysis on the target genes of differentially expressed miRNA. The expression of miRNA differential genes in blood samples stored at different times was verified by qRT-PCR. Next, we used ELISA and qRT-PCR to verify the expression of IL-1β, IL-6, IL-12 and TNF-α in blood at day 1 and day 42. In addition, *in vitro*, we transfected macrophages with overexpressed miRNA, and the effects of overexpressed miRNA on macrophage polarization and the release of inflammatory factors were verified by flow cytometry and qRT-PCR and ELISA.

Results: This study combined bioinformatics analysis and experiments to discover the differentially expressed miRNAs in long-term stored blood. The results showed that compared to fresh blood samples, the inflammatory factors were significantly doubled by ELISA, as well as the higher mRNA expression at 42 day. Experimentally verified that miR-33a-5p promoted the M1 type macrophage polarization and increased the release of related inflammatory factors through PPARα/ACC2/AMPK/CPT-1a axis regulation.

Conclusions: This study elucidates a potential mechanism of inflammatory factor accumulation in long-term stored blood, providing a theoretical basis and a potential target to prevent transfusion-related adverse reactions.

## INTRODUCTION

Although blood transfusion is life-saving in various clinical scenarios, patients are at risk of developing significant post-transfusion complications [1, 2]. Despite reassuring evidence from randomized controlled trials (RCTs), new literature is constantly emerging regarding potential clinical sequelae (other than mortality) from blood transfusions [3–5]. There are potential etiological links between storage lesions and adverse outcomes following transfusion. Retrospective observational studies have revealed that transfusion of long-term-stored blood leads to poor clinical results including a higher incidence rate of multiple organ failure, nosocomial infection, and mortality [5–7]. These unfavorable outcomes are thought to be partly due to the immunosuppressive effects of blood transfusion. In long-term-stored blood, cytokines and inflammatory cellular components are released into the plasma, resulting in gradual biochemical and morphological changes [8]. Cytokine production can occur in whole blood samples within 2 h of blood collection [9]. Extending the duration of storage can result in significant increase in the release of inflammatory cellular components [8, 10, 11]. The RBCs used for transfusion are refrigerated in a preservation solution, thus prolonging their shelf life. The duration of storage is dependent on how they are collected. The longest is 42 days for example in the US, but some products have shorter storage durations in different jurisdictions [12]. The RBCs undergo many physical and chemical changes during cold storage. These changes, collectively referred to as RBC storage lesions, affect the quality and function of the RBCs, diminishing the *in vivo* survival of transfused RBCs.

MicroRNAs (miRNAs), a group of small non-coding RNAs (approximately 19-22 nucleotides), regulate post-transcriptional gene expression by inhibiting translation or inducing target-specific messenger RNA (mRNA) [13]. These miRNAs participate in various physiological and pathophysiological processes, including immunity, cancer cell proliferation, and drug resistance [14]. Each miRNA targets multiple mRNAs, either alone or in combination with other miRNAs, thus miRNAs can regulate complex gene expression regulatory networks [15]. In blood, miRNAs, are commonly referred to as circulating miRNAs. Some studies suggested a relationship between miRNAs and the physiological state of the RBCs [16, 17]. Therefore, RBC miRNAs are considered appropriate surrogates for RBC storage lesions. The miRNAs could be potential predictors, indicating the safety and efficacy of blood products [18]. miR-33a-5p plays a crucial role in regulating cholesterol and lipid metabolism and is associated with their host genes, and the bioinformatics website further validated the miR-33a-5p targeting fork box K2 (FOXK2) and participated in the PI3K/AKT/mTOR signaling pathway through *in vitro* experiments [19, 20]. In addition, the expression level of miR-33a-5p in the circulation is disordered in diabetes patients and pre-diabetes patients, indicating that miRNA has potential diagnostic use in the detection of type 2 diabetes [21]. However, there have been no reports on the role of miR-33a-5p expression in circulation in regulating immune inflammation in stored blood.

Peripheral blood mononuclear cells and macrophages play a pivotal role in blood immune regulation. In this study, differentially expressed miRNAs in long-term-stored blood were identified by dataset analysis, and our results were combined with the findings from blood samples. The role of miRNAs in macrophage polarization was also validated through *in vitro* experiments, and the underlying mechanisms of inflammatory factor accumulation and transfusion-related adverse reactions in long-term-stored blood were determined.

## MATERIALS AND METHODS

### Gene expression data analysis

Gene expression data was obtained from miRNA analysis of stored RBC using the microarray data assay. The time-changing miRNAs in RBC were identified based on the publicly available Gene Expression Omnibus data base (GEO) (GSE114990) in the National Center of Biotechnology Information (NCBI). The time-gene sequence differential gene-based software package ‘ImpulseDE2’ was used in the R language. The R package ‘miRNetR’ was used to predict the target genes of miRNAs, and the Kyoto Encyclopedia of Genes and Genomes (KEGG) was used to perform enrichment analysis of the target genes. The results were visualized.

### Function annotation and gene set enrichment analysis

Differentially expressed genes (DEGs) were identified using the limma package. Gene ontology (GO) is a community-based bioinformatics resource that includes biological processes (BP), cell components (CC), and molecular functions (MF). KEGG is a knowledge base for the systematic analysis of gene functions that links genomic information with higher-order functional information. GO and KEGG enrichment results were generated by the R packages “ggplot2,” “enrichplot,” “clusterProfiler,” and “GOplot” for the purpose of analysis. The statistical algorithm (Fisher’s exact test) was used to find out which specific functional items a group of genes was most related to each item in the analysis results corresponds to a statistical value P-value to indicate the significance. The smaller the *P*-value is, the greater the relationship between the item and the input gene is. Gene Set Enrichment Analysis (GSEA) was performed to compare the samples by GSEA software (version 4.0.3). Functional annotations with a | log2 FC | > mean ± 2SD and *P*-value < 0.05 were considered statistically significant.

### Blood collection

Cold-stored, low-titer, O-positive, non-leukoreduced whole blood units were obtained from 20 healthy donors by our regional blood bank, and informed consent was obtained from each donor and acquired hospital ethics approval (Blood collection batch No: ISFX21012166, ISFX21012488). Whole blood (400 mL) was collected in citrate phosphate dextrose (CPD; 3 mg/mL citric acid, 26.30 mg/mL sodium citrate, 2.22 mg/mL monobasic sodium phosphate, and 25.51 mg/mL dextrose) and stored as 1.5 mL aliquots in 1.7 mL blood bag catheter (a catheter filled with whole blood) at 4° C for up to 42 days. A separate aliquot of fresh whole blood from the same donor was used to generate standard packed RBC units. Packed RBC units were generated via centrifugation at 300 g for 7 min after which the supernatant containing the platelets was discarded. Subsequently, the blood was subjected to centrifugation at 1000 g for 15 min and the supernatant containing the buffy coat was discarded. Additive Solution-3 (AS-3; 0.42 mg/mL citric acid, 5.88 mg/mL sodium citrate, 2.76 mg/mL monobasic sodium phosphate, 4.10 mg/mL sodium chloride, 10 mg/mL dextrose, and 0.30 mg/mL adenine) was added to the remaining erythrocytes in a ratio of 2:9 and stored as 1.5 mL aliquots in a 1.7 mL microcentrifuge tubes at 4° C for up to 42 days.

### Human THP-1 cell culture

THP-1 cells (human acute monocytic leukemia cell line) from American Type Culture Collection (ATCC, USA) were cultured in RPMI-1640 media (Invitrogen, 11875, USA) supplemented with 10% heat-inactivated fetal bovine serum (FBS) and 0.05 mM 2-mercaptoethanol (Sigma-Aldrich, M6250, USA) and incubated at 37° C in a 5% CO_2_ incubator. Macrophages were obtained after 72 h of THP-1 cell culture in RPMI-1640 media supplemented with 80 nM phorbol 12-myristate 13-acetate (PMA, MedChemExpress, HY-18739, China).

### Plasmid generation and cell transfection

Hsa-miR-33a-5p mimics (Mimic; #B01001, sense: 5′-GUGCAUUGUAGUUGCAUUGCA-3′, with modified mature miRNA strand: 2 phosphorothioates at the 5’ end, 4 phosphorothioates at the 3’ end, 3’ end cholesterol group, and full-length nucleotide 2’-methoxy modification) hsa-miR-33a-5p agomir Chemical Structure), inhibitor (Int; #B04004, sense: 5′-UGCAAUGCAACUACAAUGCAC-3′, full-length nucleotide 2’-methoxy modification), and mimic negative control (Mimic-NC; #B04001, sense: 5′-UGAAUGUUGGAUCGCUUCAUG-3′) were synthesized by MedChemExpress (HY-R00703, HY-RI00703, China), and negative inhibitor control (Int-NC; sense: 5′-GCACUAUACAUGAACUCGCAA-3′) were synthesized by Thermo Fisher Scientific (AM17010, USA). The cells were then transfected using Lipofectamine™ 3000 reagent (Thermo Fisher Scientific, L3000001, USA), according to the manufacturer’s instructions. The specific transfection steps were as follows: transfection was performed when the inoculated cells were passaged to 70-90% confluence. Then we used the Opti MEM™culture medium to dilute the Lipofectamine™ 3000 reagents. Next, the Opti MEM™ was used to dilute the target plasmid with culture medium. Then the Lipofectamine™ 3000 diluent and the target plasmid diluent were in a 1:1 ratio incubated at 37° C for 15 minutes, and was added to the cells for 48 hours, and then analyzed the transfected cells.

### Enzyme-linked immunosorbent assay (ELISA)

The levels of inflammatory factors, including interleukin-1β (IL-1β), interleukin-6 (IL-6), interleukin-12 (IL-12), and tumor necrosis factor-α (TNF-α), were determined using a commercial ELISA kit (Elabscience, E-EL-H0149c, E-EL-H0102c, E-EL-H0150c, and E-EL-H0109c, China). All experiments were conducted according to the manufacturer’s protocol. Briefly, for the treatment of blood sample, we used a centrifuge tube containing anticoagulant to conduct centrifugation (centrifugation condition:4° C, 2000-3000 rpm, 20 min) within 30 minutes after collecting the sample, and carefully collected the supernatant (plasma). And for the determination of the content of inflammatory factors in the cell supernatant, we collected the cell supernatant (centrifugation condition: 4° C, 600-800 rpm, 5 min) after transfected with different plasmids 24 hours. 100 μL standard working solution or sample was added into the corresponding plate well and incubated at 37° C for 90 min. The liquid in the plate was discarded and 100 μL biotinylated detection antibody working solution was added to each well and the plate incubated at 37° C for 60 min. The liquid in the plate was discarded and the plate was washed three times. Then 100 μL of Diluted Streptavidin HRP working solution (the secondary antibody) was added per well and the plate was placed in the Warm bathtub at 37° C for 30 min, after that the liquid in the plate was discarded and the plate was washed five times. Ninety microliters of substrate solution were added per well and incubated at 37° C for approximately 15 min. Termination solution (5 μL) was then added to each well, and the absorbance value was recorded at the wavelength of 450 nm using a microplate reader (Multiskan Spectrum, Thermo Fisher Scientific, USA).

### Flow cytometry

Flow cytometry staining and analysis were performed according to the manufacturer’s protocol [22]. Approximately 1 × 10^6^ cells were transferred to 1.5 mL tube, washed twice with PBS containing 10% FBS and 1% sodium azide (NaN_3_), and incubated with 10 μg/mL Anti-CD86 antibody (Abcam, ab239075, USA) [23] (one of the CD surface markers for M1-phenotype macrophage) and Anti-NOS2 (Abcam, ab283655, USA) [24] (nitric-oxide synthase 2, overexpressed in M1-phenotype macrophage) in 3% BSA/PBS in the dark for 30 min, at room temperature. The cells were then washed three times by centrifugation and resuspended in 500 μL PBS. Flow cytometry was performed using a Becton Dickinson FACSCalibur flow cytometer (San Jose, CA, USA). Data were analyzed using the FlowJo 10.4.2 software (BD Biosciences).

### Quantitative real-time PCR (qRT-PCR)

Total miRNA in whole blood was extracted using the miRcute miRNA Isolation Kit (Tiangen, DP501, China). Extracted RNA (1 μg) was reverse transcribed using a reverse transcription kit (Takara, RR047A, China). Quantitative real-time PCR was performed using gene-specific primers and an Applied Biosciences 7500 Real-Time PCR system was used for qRT-PCR. Relative expression levels of miR-33a-5p were calculated using the 2^−∆∆Ct^ method. All qRT-PCR reactions were performed in triplicate. The primer sequences used are listed in Table 1.

**Table 1:** Primer sequences used in real-time PCR analysis.

**Target gene**	**Forward primer (5’–3’)**	**Reverse primer (5’–3’)**
*miR-720*	GCGTGCTCTCGCTGGGG	GCGTGCTCTCGCTGGGG
*miR-33a-5p*	CCTCATAAGCGGTGCATTGTA	TATGCTTGTTCTCGTCTCTGTGTC
*miR-198*	GGTCCAGAGGGGAGAT	GAATACCTCGGACCCTGC
*miR-152*	CGCGCTAGCAGCACGTAAAT	GTGCAGGGTCCGAGGT
*miR-32-5p*	TATTGCACATTACTAAGCCTT	GAATACCTCGGACCCTGC
*18S*	ACACGGACAGGATTGACAGA	GGACATCTAAGGGCATCACA
*AMPK*	*TTGAAACCTGAAAATGTCCTGCT*	*GGTGAGCCACAACTTGTTCTT*
*ACC2*	*TCAGCCTACAAAACCGCCCA*	*AAGGCCGTCCACGATGTAGG*
*CPT1A*	TCCAGTTGGCTTATCGTGGTG	TCCAGAGTCCGATTGATTTTTGC
*PPAR-α*	ATGGTGGACACGGAAAGCC	CGATGGATTGCGAAATCTCTTGG
*IL-1β*	ACAAGGAGAACCAAGCAACG	GCCGTCTTTCATTACACAGG
*IL-6*	CCACTCACCTCTTCAGAACGAAT	CCTCTTTGCTGCTTTCACACAT
*IL-12*	ACCCTGACCATCCAAGTCAAA	TTGGCCTCGCATCTTAGAAAG
*TNF-α*	GGTATGAGCCCATCTATC	GCAATGATCCCAAAGTAG
*GAPDH*	GCACCGTCAAGGCTGAGAAC	ATGGTGGTGAAGACGCCAGT

### Statistical analysis

All data were analyzed using GraphPad Prism 7.0 (GraphPad Software, CA, USA). All data were presented as mean ± standard deviation (SD) of at least three independent experiments. Student’s *t*-test and one-way analysis of variance (ANOVA) were used to determine the statistical significance for comparisons of two or more groups. Pearson correlation was performed for fold-change in level of miR-33a-5p and expression level of inflammatory factors (IL-1β, IL-6, IL-12, and TNF-α). For all analyses, differences were considered statistically significant at *P* < 0.05.

## RESULTS

### Differential expression of miRNA in long-term-stored RBC

**Figure 1:**
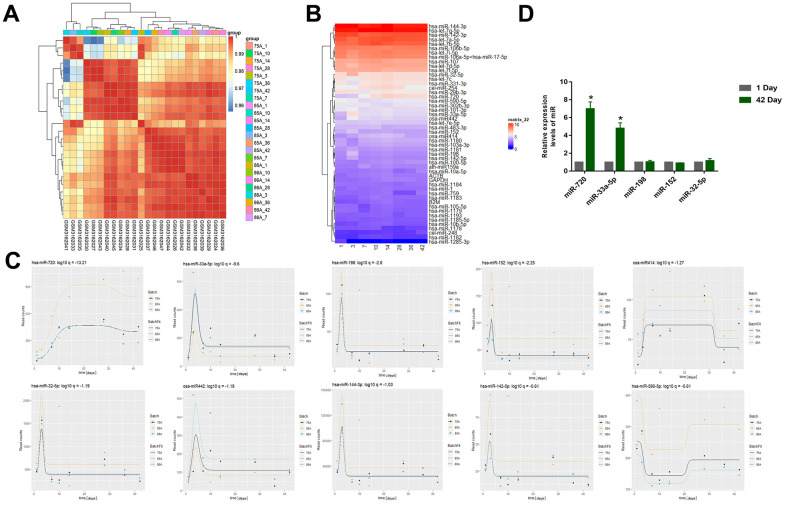
(**A**) Correlation Heatmap of samples (GSE114990). (**B**) Heatmap of miRNA expression (GSE114990) (The abscissa represents days of storage). (**C**) The top 10 miRNAs that changed significantly with storage time were miR-720, miR-33a-5p, miR-198, miR-152, miR414, miR-32-5p, miR442, miR-144-3p, miR-142-5p, miR-590-5p. (**D**) The qRT–PCR to examine the top five miRNAs using qRT-PCR of the RBC samples we collected from the donors. All data are means ± SD; n = 3 (^*^P<0.05).

Differences in stored blood RBC miRNAs were determined by gene expression data analysis and significantly changed miRNAs with longer storage times were determined by timing analysis (GSE114990). The relatedness of the different samples in the dataset were evaluated for subsequent analyses (Figure 1A). The differences in the miRNA expression levels between samples in the different groups was also demonstrated (Figure 1B). The top ten differentially expressed miRNAs were miR-720, miR-33a-5p, miR-198, miR-152, miR414, miR-32-5p, miR442, miR-144-3p, miR-142-5p, miR-590-5p (Figure 1C). Some metabolic substances gradually increase in the first two weeks under the action of oxidative stress during blood storage and significantly decrease after 14 days [25, 26], which was similar to our results. They reached a peak on the 10th day and then decreased rapidly. Then we validated the expression of the top five miRNAs using qRT-PCR of the RBC samples we collected from the donors (Figure 1D). The results showed that only miR-720 and miR-33a-5p levels were significantly elevated. However, over time, the level of miRNA in the circulation will significantly degrade, while the level of miR-720 did not significantly decrease and remained higher than the normal group after 10 days, indicating that it was not a conventional miRNA. Therefore, we chose miR-33a-5p as a follow-up study.

### More inflammatory factors are accumulated in long-term-stored blood

**Figure 2:**
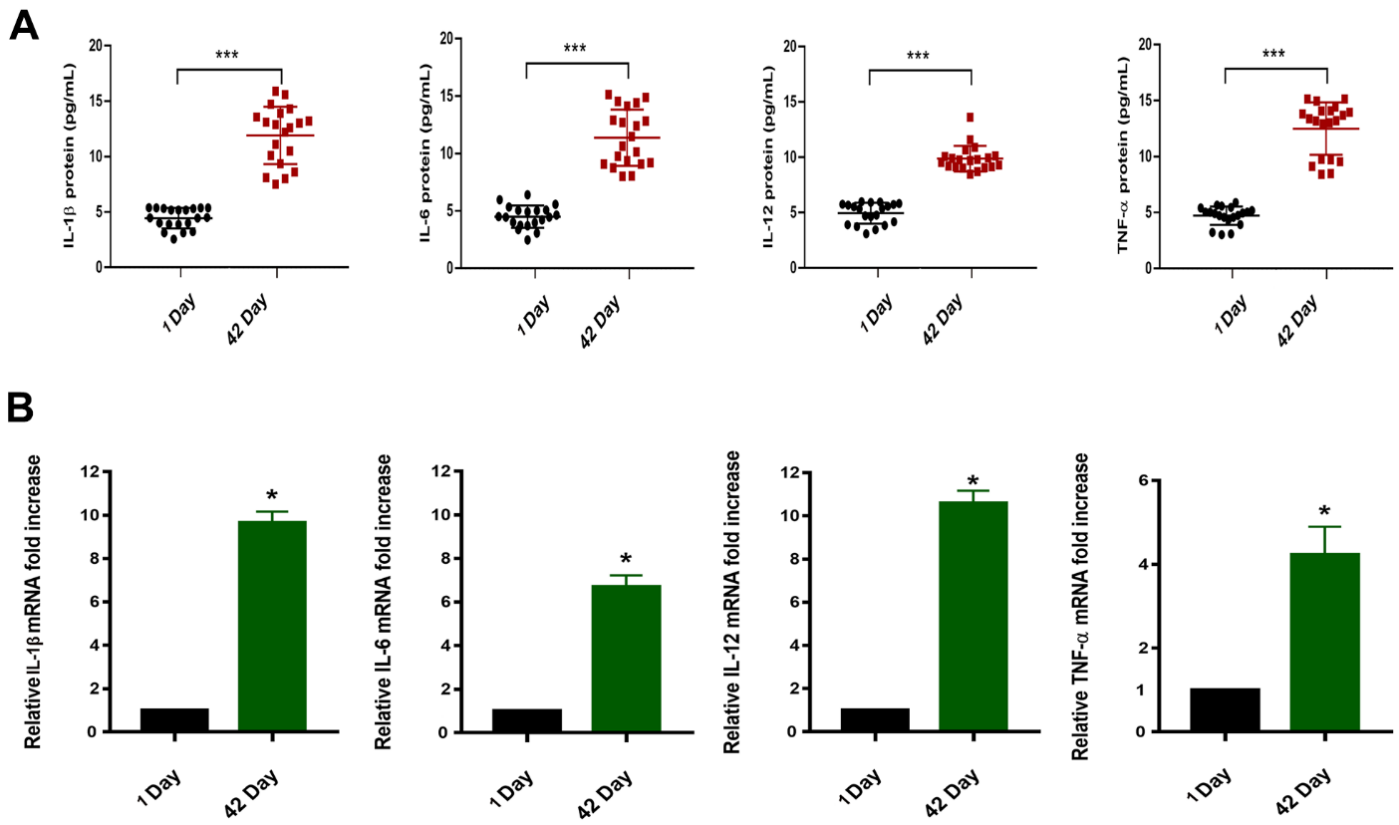
(**A**) Protein concentrations of IL-1β/IL-6/IL-12/TNF-α in the plasma of blood samples (n=20,^****^P<0.001). (**B**) Relative mRNA expression in blood samples of 1 Day and 42 Day (^*^P<0.05).

ELISA and qRT-PCR experiments were performed on the collected whole blood samples of the day 1 and the day 42. We first evaluated fold-change in level of miR-33a-5p and expression level of inflammatory factors (IL-1β, IL-6, IL-12, and TNF-α) typically released or overexpressed upon the macrophage M1 type activation through Pearson correlation analysis, which proved that there was correlation between them (Table 2). The results showed that the blood stored for a long period of time (day 42), compared to fresh blood samples, accumulated the more protein levels (increased 2-3 times) (Figure 2A) as well as the high mRNA expression (increased 2-5 times) (Figure 2B). These inflammatory factors are typically released or expressed upon macrophage M1 type activation [27]. Therefore, we hypothesized that the differential expression of miRNAs was correlated with M1-like polarization of macrophages in blood that was stored for long periods of time.

**Table 2:** Statistical analysis of correlations between levels of miR-33a-5p and inflammatory cytokines.

		**qIL-6**	**qIL-12**	**qTNF**	**qIL-1a**
Unadjusted (n=20)	Pearson Correlation with exp(ΔΔCT _miR-33a-5p_)	.288	-.102	-.364	.081
	Sig. (2-tailed)	.218	.168	.115	.733
Adjusted* (n=18)	Pearson Correlation with exp(ΔΔCT _miR-33a-5p_)	-.415	.096	-.600^**^	-.066
	Sig. (2-tailed)	.016	.003	.008	.795

*The 2 outliers exp(ΔΔCT _miR-33a-5p_) are removed.

qIL-6 = IL6_Day 42_/IL6_Day 1_, qIL-12 = IL-12_Day 42_/ IL-12_Day 1_, qTNF = qTNF_Day 42_/ qTNF_Day 1_, qIL-1a = qIL-1a_Day 42_/ qIL-1aDay 1.

### MiR-33a-5p drives macrophage polarization toward M1 type and promotes the release of inflammatory factors

**Figure 3:**
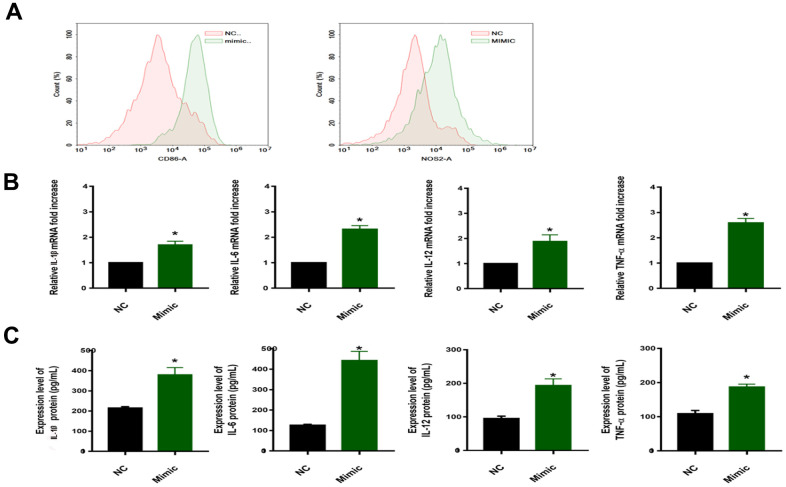
(**A**) Flow cytometric examination of CD86/NOS2 expression of macrophages. (**B**) qRT–PCR for examination of PPARα/ACC2/AMPK/CPT-1a mRNA expression (^*^P<0.05). (**C**) Protein concentrations of IL-1β/IL-6/IL-12/TNF-α in the media of cells from different groups (^*^P<0.05). NC: normal macrophages group; Mimic: macrophages transfected with the miR-33a-5p mimic. All data are means ± SD; n = 3.

To verify this hypothesis, the Flow cytometry and qRT-PCR were performed on macrophages (induced by THP-1 with PMA) transfected with the miR-33a-5p mimic. Polarized macrophages were characterized by their differential expression of CD surface markers and cytokine secretion. We selected CD86 and NOS2 (generally considered as regulatory genes for M1 polarization of macrophages) [23, 24] to judge the effect of miR-33a-5p on macrophage polarization. Compared with non-transfected normal macrophages (NC), macrophages transfected with the miR-33a-5p mimic (Mimic) expressed more CD86 and NOS2, resulting in the M1 type polarization (Figure 3A), as well as the mRNA (increased 1-2 times) (Figure 3B) and the protein levels of the release of inflammatory factors IL-1β, IL-6, IL-12, and TNF-α (increased 1-4 times) (Figure 3C).

### miR-33a-5p drives macrophage polarization toward M1 type via the adipocytokine signaling pathway

**Figure 4:**
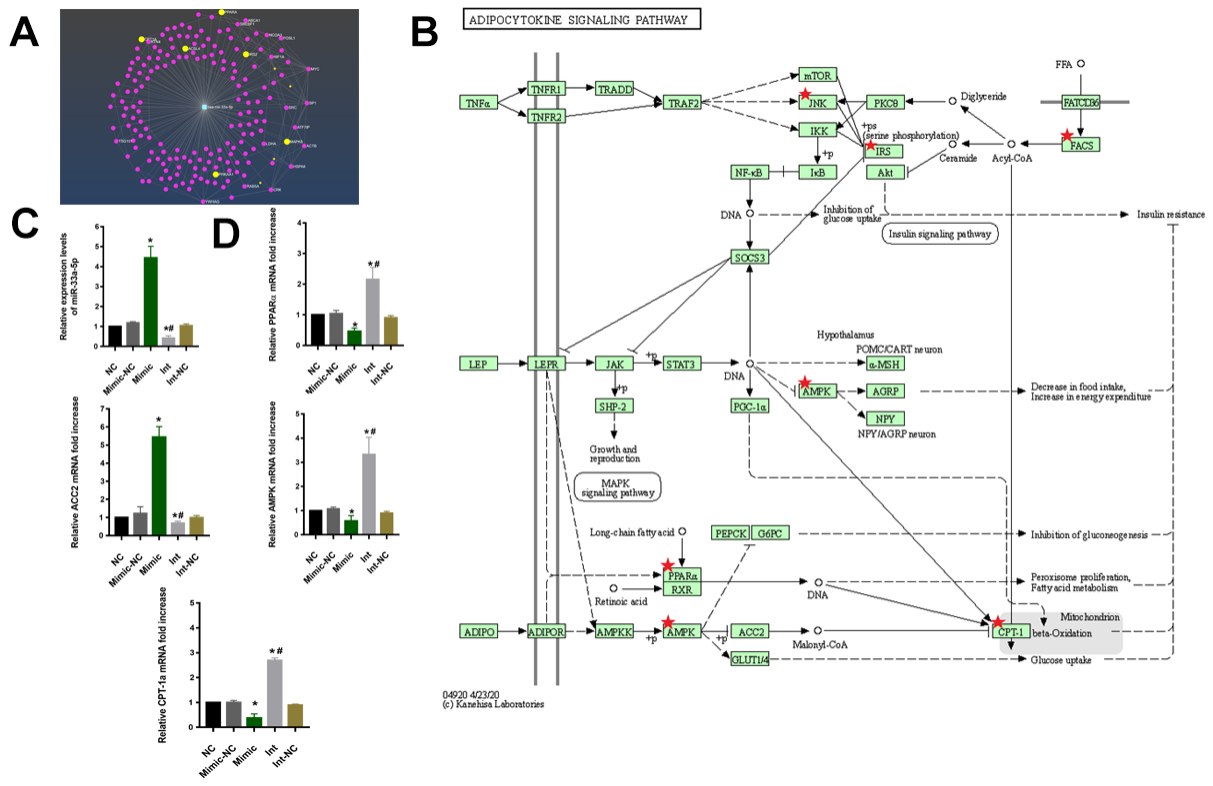
(**A**) The net plot of mir-33a-5p and its regulated mRNA target genes. (**B**) The signaling pathway of KEGG showed that the target mRNA genes were enriched in the adipocytokine signaling pathway and the PPARα/AMPK/CPT-1a pathway was the key signaling pathways. (**C**) qRT–PCR to examine miR-33a-5p expression. (^*^P<0.05 vs NC, *^#^P<0.05* vs Mimic). (**D**) qRT–PCR to examine PPARα/ACC2/AMPK/CPT-1a mRNA expression. (^*^P<0.05 vs NC, *^#^P<0.05* vs Mimic). NC: normal macrophages group; Mimic-NC: macrophages transfected with the mimic negative control; Mimic: macrophages transfected with the miR-33a-5p mimic; Int: macrophages transfected with the inhibitor; Int-NC:macrophages transfected with the negative inhibitor control. All data are means ± SD; n = 3.

To investigate the regulatory mechanism of miR-33-5p in macrophage M1 polarization, the regulated target mRNA genes of miR-33a-5p were screened. The R package, and miRNetR were used for the targeted mRNA genes of miRNA (Figure 4A). And the KEGG enrichment analyses showed that the target mRNA genes were enriched in the adipocytokine signaling pathway and the PPARα/AMPK/CPT-1a pathway was the key signaling pathways (Figure 4B).

The macrophages were then transfected with the miR-33a-5p mimic (Mimic), mimic negative control (Mimic-NC), inhibitor (Int), and negative inhibitor control (Int-NC). The qRT-PCR results showed that the transfection was successful (Figure 4C). Compared with the control, the overexpression of the miR-33a-5p resulted in the suppression of PPARα/AMPK/CPT-1a as well as the elevation of ACC2 mRNA levels. In contrast, knockdown of miR-33a-5p had the opposite effect (Figure 4D).

## DISCUSSION

The effect of RBC transfusion with different storage periods on individual clinical outcomes has been an active and controversial topic in the clinical community [5]. Stored blood cells undergo progressive biochemical and morphological deterioration, which include reduced erythrocyte viability, cell size, deformability, lipid membrane composition, and release of inflammatory factors [28]. Importantly, RBCs storage lesions are considered the cause of many adverse effects following transfusions [29]. RBCs storage also affects the immune function of the recipient. Long-term storage induced high expression levels of a variety of cytokines, including IL-6, IL-8, phospholipase A2, and superoxide anions, in plasma or whole blood samples [30].

During RBC suspension storage, various factors are continuously produced as tools for intercellular communication and molecular transportation. During storage, exosomal miRNAs in erythrocyte suspensions can promote the secretion of certain inflammatory cytokines and mediate T cell proliferation [31]. miRNAs play key roles in erythropoiesis. Many upregulated or downregulated miRNAs in erythropoiesis may not be removed or degraded but selectively remain in mature erythrocytes [32, 33]. Most miRNAs released by RBCs was stably expressed and the main source of miRNAs in the whole blood [18]. However, other components in the blood also release some miRNAs and they cannot be distinguished from miRNAs secreted from leukocytes and platelet [34]. This limits the application of miRNAs as disease biomarkers and affects their accuracy for the prediction of blood storage security and transfusion-related adverse reactions.

Therefore, a dataset of stored RBCs for analysis were selected to ensure that the resulting differential miRNAs were RBC-specific. We demonstrated, by time series analysis, that the expression levels of many miRNAs changed drastically during the first 10 days (Figure 1C). The miRNAs such as miR-33a-5p, miR-198, and miR-152 showed a rapid increase in expression levels, followed by a rapid decline, except for miR-720. The expression level of miR-720 continued to increase and a certain level was maintained which was similar to the results obtained by Yang et al. [30]. Yang et al. suggested that many cellular proteins and nucleic acids may undergo systematic degradation due to the physiological pressure experienced by RBCs during storage, and certain synthetic products in the cytoplasm can be used as stabilizers for particular selected miRNAs during storage. As observed in our study, there may be an increase in the false appearance of miRNAs during storage. Thus, the significant increase in miRNAs pre-storage is the result of the gradual stabilization of miRNAs over time during RBC storage [25]. The miR-720 was no more than a cleavage product of RBCs than a miRNA that is specifically regulated as a function of storage time. Metabolic parameters change rapidly during the first two weeks of RBC storage [26]. Thus, metabolism plays a role in RBC storage, maintaining RBC energy production during the first two weeks of metabolism; from day 14, RBCs start to produce metabolites that engage in antioxidant responses. There was a high degree of temporal overlap between the changes in these metabolic parameters and miRNAs, which can be used as biomarkers of blood storage damage. Studies have implicated miR-720 as a tRNA and have suggested removed from the miRBase [30]; and we collected RBC samples to compare the expression gaps of these miRNAs (Figure 1D), and only miR-720 and miR-33a-5p had distinct expression differences. Thus, miR-33a-5p was the focus of this study.

Macrophages represent a heterogeneous cell population with a dynamic functional state spectrum from pro-inflammatory M1 to anti-inflammatory/immunomodulatory selectively activated M2 macrophages, exhibiting significant differences in gene expression characteristics and effector functions [27, 35, 36]. The different functional phenotypes of macrophages M1 and M2 depend on the coordinated expression of various regulators that promote opposite functions. The release of chemokines IFN-γ, TNF-α, IL-1β, IL-6, IL-12 and proteases, as well as the production of reactive oxygen species (ROS) and the cell surface molecules (Cd86, INOS/NOS2) is a prominent feature of M1, however, the M2 macrophages express other molecules, including chitinase family proteins and mannose receptor type 1 CD206 [27, 37, 38]. In our studies, the whole blood samples collected for long-term storage demonstrated high expression levels of the inflammatory factors IL-1β, IL-6, IL-12, and TNF-α (Figure 2). And we found there was a significant correlation between the fold-change in level of miR-33a-5p and the expression level of the inflammatory factors through the Pearson correlation. Therefore, we hypothesized that long-term storage of blood promotes the release of inflammatory factors by regulating M1 polarization of macrophages via miR-33a-5p.

To test this hypothesis, we transfected the macrophages with miR-33a-5p mimics, and we found the expression of the CD86/NOS2 was higher, resulting in the M1 type polarization (Figure 3A), as well as the release of inflammatory factors IL-1β, IL-6, IL-12, and TNF-α (Figure 3B, 3C). This was consistent with the result of Maryam [37], indicating that miR-33a-5p can regulate macrophage M1 polarization. The specific regulatory mechanism of miR-33a-5p in the M1 polarization of macrophages was investigated further. Firstly, the specific messenger RNA (mRNA) targets regulated by miRNA were predicted in different ways (Figure 4A), and the pathway enrichment analysis was performed on the targets. The adipocytokine signaling pathway was regulated by the miR-33a-5p multisite (Figure 4B). In the adipocytokine signaling pathway, miR-33a-5p targeted several genes such as *PPARα/ACC2/AMPK/CPT-1a*. The activation of genes such as PPARα/AMPK/CPT-1a can effectively promote lipid metabolism and alleviate inflammation-related diseases [39]. Accumulation of bioactive lipids during RBC storage has been identified as a potential source of transfusion-related adverse effects in susceptible individuals [40]. A targeted metabolomics study showed an accumulation of polyunsaturated fatty acids (PUFAs) and their oxidation products (oxylipins) in RBC units stored for 42 days. The accumulation analysis of a panel of bioactive lipids in leukoreduced and non-leukoreduced RBC units indicated that leukoreduction greatly attenuated the production of bioactive lipids [41]. Thus, it is highly likely that the adipocytokine signaling pathway is regulated by miR-33a-5p in leukocytes, which promotes lipid accumulation in long-term-stored blood samples. The presence or absence of lipids has a significant impact on macrophage biology, as macrophages have been implicated in the pathogenesis of diseases in which lipid homeostasis is disturbed [42]. In one study, CPT1A knockdown promoted the upregulation of iNOS, a pro-inflammatory marker, in macrophages [43]. Real-time qPCR was used to validate our results (Figure 4D). The miR-33 has also been implicated in regulating lipid metabolism in macrophages [44]. Transfusion-related acute lung injury (TRALI) is a major cause of adverse transfusion-related effects caused by anti-leukocyte antibodies or biological response modifiers (e.g. lipids) [45]. Various studies corroborate our findings, indicating that miR-33a-5p can be a therapeutic target for the prevention of transfusion-related adverse reactions.

In conclusion, we demonstrated that miR-33a-5p mediates the crosstalk between inflammatory factor accumulation in stored blood and macrophages. This contributed to the activation of M1 macrophages through the *PPARα/ACC2/AMPK/CPT-1a* pathway. Our study indicates that miR-33a-5p may represent a novel target for the prevention of the progression of transfusion-related adverse reactions and may be a new biomarker for evaluating the quality of RBC units.

**Figure 5:**
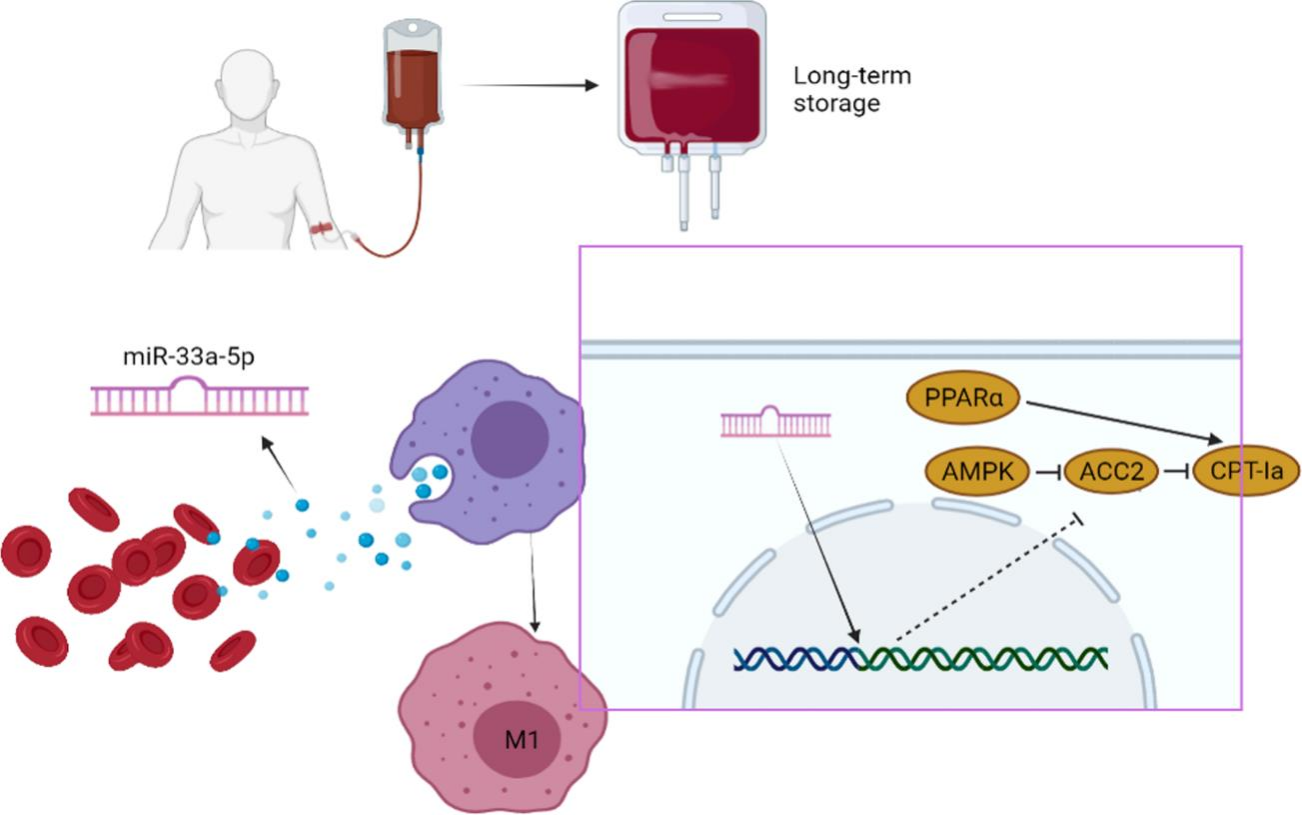
**Summary schematic of this study: miR-33a-5p mediates the crosstalk between inflammatory factor accumulation in stored blood and macrophages.** This contributed to the activation of M1 macrophages through the *PPARα/ACC2/AMPK/CPT-1a* pathway.

Furthermore, with the long-term storage, miRNAs related to a variety of immune signaling pathways accumulate in the blood. Among these, miR-33-5p can be used as a biomarker to evaluate the quality of transfused blood (Figure 5).
